# Infectious Salmon Anemia Virus Infectivity Is Determined by Multiple Segments with an Important Contribution from Segment 5

**DOI:** 10.3390/v14030631

**Published:** 2022-03-18

**Authors:** Matías Cárdenas, Sofía Michelson, Daniel R. Pérez, Margarita Montoya, Jorge Toledo, Yesseny Vásquez-Martínez, Marcelo Cortez-San Martin

**Affiliations:** 1 Molecular Virology and Pathogen Control Laboratory, Departamento de Biología, Facultad de Química y Biología, Universidad de Santiago de Chile (USACH), Santiago 9170022, Chile; matias.cardenas.p@usach.cl (M.C.); sofia.michelson@usach.cl (S.M.); yesseny.vasquez@usach.cl (Y.V.-M.); 2 Poultry Diagnostic and Research Center, Department of Population Health, University of Georgia, Athens, GE 30602, USA; dperez1@uga.edu; 3 Cell Biochemistry Laboratory, Department of Biology, Faculty of Chemistry and Biology, University of Santiago, Santiago 9170022, Chile; margarita.montoya@usach.cl; 4 Biotechnology and Biopharmaceutical Laboratory, Departamento de Fisiopatología, Facultad de Ciencias Biológicas, Universidad de Concepción, Concepción 4070386, Chile; jotoledo@udec.cl; 5 Programa Centro de Investigaciones Biomédicas Aplicadas, Escuela de Medicina, Facultad de Ciencias Médicas, Universidad de Santiago, Santiago 9170022, Chile

**Keywords:** isavirus, salmonid, reverse genetic, infectivity

## Abstract

Infectious salmon anemia virus (ISAV) is the etiological agent of infectious salmon anemia. It belongs to the genus isavirus, one of the genera of the *Orthomyxoviridae* family, as does Influenzavirus A. The ISAV genome comprises eight negative-sense single-stranded RNA segments that code for at least 10 proteins. Although some ISAV strains can reach 100% mortality rates, the factors that determine isavirus infectivity remain unknown. However, some studies suggest that segments 5 and 6 are responsible for the different degrees of virulence and infectivity among ISAV subtypes, unlike the influenza A virus, where most segments are involved in the virus infectivity. In this work, synthetic reassortant viruses for the eight segments of ISAV were generated by reverse genetics, combining a highly virulent virus, ISAV 752_09 (HPR7b), and an avirulent strain, SK779/06 (HPR0). We characterized the rescued viruses and their capacity to replicate and infect different cell lines, produce plaques in ASK cells, and their ability to induce and modulate the cellular immune response in vitro. Our results show that the majority of ISAV segments are involved in at least one of the analyzed characteristics, segment 5 being one of the most important, allowing HPR0 viruses, among other things, to produce plaques and replicate in CHSE-214 cells. We determined that segments 5 and 6 participate in different stages of the viral cycle, and their compatibility is critical for viral infection. Additionally, we demonstrated that segment 2 can modulate the cellular immune response. Our results indicate a high degree of genetic compatibility between the genomic segments of HPR7b and HPR0, representing a latent risk of reassortant that would give rise to a new virus with an unknown phenotype.

## 1. Introduction

Infectious salmon anemia virus is the etiological agent of infectious salmon anemia. It belongs to the family *Orthomyxoviridae*, to which the Influenza A virus also belongs, a virus widely used as a reference model for the study of ISAV. Isaviruses are enveloped and pleomorphic, with an average viral particle diameter of 100 nm [1]. Its genome is composed of eight negative-sense single-stranded RNA segments (vRNA) [2] that code for at least 10 proteins [3]. Segments 1, 2, 3, and 4 code for the polymerase basic protein 2 (PB2) [4], the polymerase basic protein 1 (PB1) [5], the nucleoprotein (NP) [6], and the polymerase acidic protein (PA) [6], respectively. These four proteins are associated with the viral genome, and form the ribonucleoprotein complexes (vRNP) that are the minimal infectious unit of the virus [7]. Segments 5 and 6 encode the viral surface proteins Fusion (F) and hemagglutinin-esterase (HE), respectively. F promotes the fusion of the viral and the endosomal membrane [8], and HE presents both receptor binding and destroying activity [9]. Segment 7 has a single ORF and, through alternative splicing, produces two mRNAs that code for the non-structural proteins, NS1 and NS2 (NEP) [10], that participate in the interferon alfa (IFN-α) downregulation [11] and in the exportation of viral vRNPs [12] from the nucleus, respectively. Finally, segment 8 has two ORFs that code for two proteins, the M1 protein, which forms the viral capsid [13], and the second polypeptide (S8ORF2), which has two nuclear localization signals, ion-channeling activity, and the capacity to bind to RNA and modulate the expression of IFN-α [11].

Among the ISAV genome, segment 6 is the most variable [2], especially in the highly polymorphic region (HPR), a unique feature among orthomyxoviruses. To date, more than 30 HPR variants in length have been reported [14]. HPR viruses are considered avirulent, since they infect fish, but do not produce clinical symptoms of the disease [15], and interestingly they carry a full-length HPR region. On the other hand, virulent isavirus strains possess a deleted HPR region, termed HPRΔ, for which different degrees of virulence have been linked to the length and position of the deletion [16]. The most important HPRΔ affecting Chilean salmon farming is the HPR7b genotype. This strain (ISAV 752_09) was isolated and fully sequenced by our laboratory [13], and caused the Chilean salmon industry crisis in 2007 [15]. On top of that, this strain has the shortest HPR region ever described, and is highly virulent [17], reaching mortality rates close to 100%. 

To date, no explanation has been found for the relationship between the HPR length and the virulence of isavirus strains. In vitro studies have shown that co-expression of HE with deleted HPRs promotes the activity of the F protein, probably because full-length HPRs hide the cleavage site of the fusion protein, impeding its activation [18]. Additionally, a recent study described that the amino-termini of the fusion protein (F1) can interact with a cellular membrane receptor and modulates HE-mediated adsorption [19], suggesting that the HPR region is closely related to the activity of the F protein. However, the basis and implications of this interaction remain unknown.

On the other hand, segment 5 also presents genetic variability, and a total of five insertions in the cleavage site [20] have been described (IN1 through IN5). As in influenza A, these insertions are likely to provide new proteolytic cleavage sites, which increases the probability that any given cell can activate the F protein to start the infective cycle [13]. Moreover, the capacity of the fusion protein to interact with the HE HPR region may depend on the previously mentioned insertions [21], although the molecular basis of this phenomenon has not been clarified. Taken together, these antecedents strongly suggest that ISAV infectivity is primarily determined by segments 5 and 6 genotypes, and no other genomic segments have yet been associated with ISAV virulence and infectivity [20].

Quite the opposite, for influenza A, several genomic segments contribute to the virus infectivity and virulence phenotype. As was described above for isavirus, the homolog segments of influenza A virus that code for the two surface proteins hemagglutinin (HA) and neuraminidase (NA) have been described as the major infectivity determinants [22], with reports showing that mutations in HA can affect its glycosylation, which directly impacts the virus receptor-binding activity [23] and, consequently, the viral absorption step. Additionally, mutations in the NA protein can modulate the mechanism through which the HA protein, which also has a fusion domain, is processed by cellular proteases [24]. On top of that, specific mutations in the PB1 segment can increase the capacity of the influenza virus to induce cell death [25], and mutations in the PB2 segment can expand the host range of the virus, promoting the rise of zoonotic viruses. This effect can also be achieved through modifications in the HA gene [26].

Furthermore, variation in the NP protein can confer resistance to the antiviral cellular immune responses such as the Interferon-stimulated genes (ISGs, e.g., ISGylation) and the antiviral Mx protein [27]. Finally, the NS segment, especially the NS1 protein, plays a significant role in influenza A infectivity, since it functions as an IFN-α expression repressor; therefore, if the sequence of this protein changes, it can positively or negatively affect the immune response avoidance [28]. With this overwhelming evidence that numerous segments in influenza A participate in the virus infective phenotype, it cannot be discarded that something similar occurs in isavirus. However, no studies have evaluated the role of each segment in infectivity to date.

The objective of this work was to analyze the contribution of each isavirus genomic segment to the in vitro infectivity. To this end, a set of reassortant viruses were generated using an ISAV 752_09 (HPR7b) backbone and exchanging each segment for its ISAV SK779/06 (HPR0) homolog. Our results show that all the segments play a role in the replicative phenotype of the virus, segment 5 being one of the most important. This segment also seems to be involved in the ability of the virus to produce the cytopathic effect, since its introduction into an HPR0 backbone makes the virus able to produce plaques. Moreover, segments 5 and 6 give together confer the virus the capacity to infect and replicate in CHSE-214 cells. Our results also show a high degree of genetic compatibility between segments of the virulent HPR7b and the avirulent HPR0 genotypes, which suggests a latent risk of natural reassortment in vivo and, consequently, new ISAV outbreaks because of the high HPR0 prevalence.

## 2. Materials and Methods

### 2.1. Cell Culture

The Atlantic salmon cell line ASK (CRL-2747, ATCC) was used to propagate the viruses and perform most of the replication studies, while salmon embryo cells (CHSE-214; 91041114, ECACC) and rainbow trout gonad cells (RTG-2; 90102529, ECACC) were used to evaluate the reassortant viruses host range. ASK and RTG-2 cells were cultured in Leibovitz medium (L-15, HyClone, Logan, UT, USA) supplemented with 50 µg/mL of gentamicin (HyClone, Logan, UT, USA), 10% fetal bovine serum (FBS; Corning Cellgro, Mediatech, Scottsdale, AZ, USA), and 4 mM of L-glutamine (Corning Cellgro, Mediatech, Manassas, VA, USA).

CHSE-214 cells were cultured in minimal essential medium (MEM, Sigma, St. Louis, MO, USA), supplemented with 2 mM glutamine (Gibco, Grand Island, NY, USA), 10% non-essential amino acids (Corning Cellgro, Mediatech, Manassas, VA, USA), 100 UI of penicillin (Sigma, St. Louis, MO, USA), and 100 µg/mL streptomycin (Sigma, St. Louis, MO, USA). All of the cell lines were cultured at 15 °C without CO_2_, and culture media was replaced once a week.

### 2.2. Generation of Reassortant Recombinant Viruses (rISAV)

Reassortant viruses were generated using a plasmid-based reverse genetic system previously described by Toro-Ascuy et al., 2015 [29]. Briefly, 2.5 × 10^4^ cells/cm^2^ were seeded on a 12-well plate and incubated at 15 °C for 24 h, or until they reached 80% confluence. Subsequently, ASK cells were transfected with 1 µg of each pSS-URG-S1/S8 plasmid (Table 1) and 250 ng of each expression vector (pCDNA3.1/PB1, pCDNA3.1/PB2, pCDNA3.1/PA, and pCDNA3.1/NP) using a Fugene 6 (Promega, Madison, WI, USA): DNA ratio of 3:2. To do so, the transfection reagent was diluted in serum-free L-15 media, then added to the plasmid mixture, and this transfection mix was incubated for 30 min at room temperature. Afterward, the transfection mixture was added dropwise onto the cells, and then the cells were incubated at 15 °C for 4 h. After the incubation, the transfection mixture was discarded, and cells were washed three times with PBS. Finally, 1 mL culture media was added, and cells were maintained for seven days at 15 °C.

As a negative control, one transfection was performed with all the plasmids used to generate rISAV, except for the pCDNA3.1/NP plasmid (NP control). Due to this, no virus was expected to be rescued from this control.

### 2.3. rISAV Propagation in ASK Cells

After transfection, all viruses were propagated in ASK cells previously seeded on a 12-well plate at a density of 2.5 × 10^4^ cells/cm^2^, and incubated for 24 h at 15 °C before infection. The transfection supernatant was diluted tenfold in a serum-free L-15 medium. Afterward, cells were washed twice with PBS, and 500 µL of virus dilution was added. The infection was incubated for 4 h at 15 °C. Subsequently, the supernatant was discarded, the cells were washed three times with PBS, and 1 mL of culture media with 10% FBS was added. The plates were maintained for seven days at 15 °C, and the process was repeated until obtaining three blind passages [30].

### 2.4. RT-PCR and Sequencing

For viral detection and genetic analysis, rISAV RNA was purified from either the supernatant of transfected or infected ASK cells using the E.Z.N.A Total RNA kit I (Omega Bio-tek, Norcross, GA, USA), according to the manufacturer’s instructions, and contaminant plasmid-derived DNA was eliminated by treatment with an RNase-free DNase (RQ1, Promega, Madison, WI, USA). 

The two-step RT-PCR was performed using the specific primers listed in Table 2. First, cDNA was made by mixing 5 µL of RNA, 10 μM of each primer, and 3 µL of nuclease-free molecular grade water. This mixture was incubated for five minutes at 90 °C. Immediately after, reactions were put on ice, and 5 µL of 5× reaction buffer, 1 µL of M-MLV (Promega, Madison, WI, USA), 0.5 µL of RNase Out (40 U/µL, Thermo Scientific, San Jose, CA, USA), 1 µL of dNTPs 10 µM, and 7.5 µL of nuclease-free molecular grade water were added. Finally, all reactions were incubated for one hour at 42 °C.

After cDNA synthesis, the PCR reaction was performed by preparing the following master mix: 5 µL of RT product, 12.5 µL of GoTaq Green Master Mix (Promega, Madison, WI, USA), 1 µL of each primer 10 μM (same primers used for cDNA synthesis), and 5.5 µL of nuclease-free molecular grade water. The PCR products were tested on 2% agarose gels and purified using a Wizard SV Gel and PCR Clean-Up System kit (Promega, Madison, WI, USA), following the manufacturer’s instructions.

Purified PCR products were sent to Macrogen Inc., Seoul, South Korea, for Sanger sequencing using a 3730XL genetic analyzer (Applied Biosystems, Bedford, MA, USA). Sequencing was performed using the same primer pairs used for each PCR reaction.

### 2.5. ISAV Detection by Indirect Immunofluorescence

ASK cells CHSE-214 cells were seeded at a density of 2.5 × 10^4^ and 1 × 10^5^ cells/cm^2^, respectively, on a 12-well plate and incubated at 15 °C for 24 h, or until they reached 80% confluence. Cells were infected using the supernatant from the third blind passage of each virus as described above, and infected cells were maintained for five days at 15 °C. After the incubation, all samples were processed for indirect immunofluorescence, following the protocol described by Rivas-Aravena et al., 2011 [31]. Briefly, at five days post-infection (dpi), cells were washed three times with PBS (2 min each), fixed with 4% paraformaldehyde for 30 min, and permeabilized with Triton X-100 0.3% (Calbiochem, Burlington, MA, USA) in PBS for 10 min. Then, cells were incubated for one hour in blocking buffer (3% BSA and 0.1% Triton X-100 in PBS), and a primary anti-ISAV NP antibody (clone 2C2/H4, Grupo Bios Chile) diluted 1:500 in blocking buffer was added. After 1 h of incubation, cells were washed three times with PBS, and incubated for 1 h with an Alexa Fluor 594-conjugated secondary antibody (Invitrogen, Springfield OR, USA) diluted 1:1000 in blocking buffer. Nuclear staining was performed by incubating cells with 0.5 μg/mL with 4′,6-diamidino-2-fenilindol (DAPI, Thermo Scientific, Orlando, FL, USA) in blocking buffer for 1 h. Finally, the cells were washed five times with PBS, and mounted on glass slides with 1,4-diazabicyclo(2,2,2)octane (DABCO) (Sigma, St. Louis, MO, USA). Sample imaging was performed using a Zeiss LSM 800 confocal microscope at the Confocal Microscopy Unit of the Universidad de Santiago de Chile.

### 2.6. Transmission Electron Microscopy (TEM)

Samples for EM were processed as described by Toro-Ascuy et al., 2015 [29]. Briefly, ASK cells were seeded at a density of 2.5 × 10^4^ cells/cm^2^, and incubated at 15 °C until reaching 90% of confluence. Then, cells were infected using a 1:10 dilution of the supernatant from the third blind passage of each virus. At 4 dpi, cells were fixed with 2.5% glutaraldehyde in 0.1 M of cacodylate buffer, pH 7.2, for 6 h at room temperature. Subsequently, cells were washed with cacodylate buffer for 18 h at 4 °C, fixed with 1% osmium tetroxide for 90 min, submerged in distilled water, and stained with 1% uranyl acetate for 1 h. Stained samples were then dried in batteries with increasing acetone concentrations (50, 70, 95, and 100%) for 20 min at each concentration. The dehydrated samples were embedded in an epoxy-acetone resin (1:1) overnight, and then submerged in a pure EPON resin polymerized at 60 °C for 24 h. Afterward, cells were processed for ultrathin sectioning on a Sorvall MT-5000 ultramicrotome. Ultrathin cuts were mounted on copper grills, and stained with 4% uranyl acetate in methanol for 2 min. Finally, cells were observed in a Philips Tecnai 12 transmission electron microscope at 80 kV at the Advanced Microscopy Unit of the Pontificia Universidad Católica de Chile.

### 2.7. Infection Kinetics

ASK and RTG-2 cells were seeded at a density of 2.5 × 10^4^ cells/cm^2^, while CHSE-214 cells were seeded at a density of 1 × 10^5^ cells/cm^2^ on a 96-well plate, and incubated at 15 °C for 24 h. Subsequently, they were infected at a multiplicity of infection (MOI) of 0.1, and time points were collected at 0, 3, 5, and 7 dpi. Cells from the 7-dpi time point were also used to assess cell viability, as described below. 

RNA from each time point was extracted as described above, and purified RNA was retrotranscribed using the specific primers F5 (CTACACAGCAGGATGCAGATGT) and R5 (CAGGATGCCGGAAGTCGAT) [32]. The resulting cDNA was used to amplify the viral segment 8 by qRT-PCR, which was performed by preparing the following master mix: 1 µL of primer F5 10 µM, 1 µL of primer R5 10 µM, 10 µL of Takyon Master mix (EUROGENTEK, Leuven, Belgium), 6 µL of molecular grade nucleases-free water, and 2 µL of cDNA. The amplification was carried out under the following conditions: 3 min of hot start at 95 °C, followed by 40 cycles of 5 s at 95 °C, and 10 s at 60 °C. Finally, the absolute quantification of rISAV segment 8 was performed using a plasmid standard curve of known concentration.

As a control, we performed infection kinetics in the presence of ribavirin, a well-known ISAV inhibitor. The same procedure described above was used, but after 4 h of incubation, the media added to the cells was supplemented with 0.5 µM ribavirin in DMSO. 

### 2.8. Cell Viability Assay

At 7 dpi, cells were washed three times with PBS, supplemented with 5 mg/mL of 3-(4,5-dimethylthiazol-2-yl)-2,5-diphenyltetrazolium bromide(MTT, Sigma, St. Louis, MO, USA) in culture media, and incubated for 4 h in the dark. Then, the supernatant was discarded, and 100 µL of dimethyl sulfoxide (DMSO, Sigma, Marlborough, MA, USA) was added. Subsequently, the DMSO was transferred to a new 96-well plate, and absorbance was measured at 570 nm in a microplate reader (InfinitePro 200, TECAN, Männedorf, Switzerland). The cell viability percentage was determined as a function of the absorbance of non-infected control cells.

### 2.9. Plaque Assay

The assay was based on what was described by Castillo-Cerda et al., 2014 [33]. Briefly, ASK cells were seeded on a 12-well plate at a density of 7.8 × 10^4^ cells/cm², and incubated at 15 °C until reaching a 100% confluence. Then, serial 10-fold dilutions of viral inoculum from the first blind passage of each virus in serum-free L-15 media were prepared. Culture media was removed from the plates, cells were washed three times with PBS, and infected with 500 µL of the corresponding virus dilution. The infection was incubated for 4 h at 15 °C, and, afterward, the supernatant was discarded. Cells were washed three times with PBS, and immediately overlaid with 2 mL of 0.5% low melting point agarose (Winkler, Santiago, Chile) in 10% FBS L-15 culture media. The infection was incubated for 18 days at 15 °C. After incubation, cells were fixed with 37% formaldehyde in PBS for 2 h at room temperature under gentle agitation, agarose was removed, and plaques were stained with 1% crystal violet in PBS for 1 h.

### 2.10. Early and Late Adsorption and Uptake Evaluation

To assess differences in early and late adsorption and uptake events, ASK cells were seeded on a 12-well plate at a density of 2.5 × 10^4^ cells/cm^2^, and incubated at 15 °C until reaching 80% confluence.

To evaluate early adsorption and uptake differences between reassortant viruses, cells were infected at an MOI of 1 using the supernatant from the first blind passage of each virus, and then incubated for two hours at 15 °C. Following incubation, the cells were washed three times with PBS, and total RNA was extracted for further rISAV titration by qRT-PCR as described above. 

On the other hand, the same procedure described above was followed to assess late adsorption and uptake differences. Still, this time, after the 2 h incubation period, cells were washed three times with PBS, supplemented with culture media, and incubated at 15 °C for an extra hour. Subsequently, cells were washed three times with PBS, and treated with 100 µg/mL proteinase K (Sigma, Darmstadt, Germany) in PBS for 10 min at room temperature. After incubation, the cellular suspension was washed three times with PBS to discard any remaining viruses. Finally, cellular RNA was extracted, and the rISAV segment 8 copy number was determined by qRT-PCR as described above.

### 2.11. IFN-α, Mx, and ISG15 Relative Quantitation

ASK cells were seeded on a 12-well plate at a density of 2.5 × 10^4^ cells/cm^2^, and incubated at 15 °C until reaching an 80% confluence. Subsequently, cells were infected at an MOI of 0.1, and cellular RNA was extracted at 4, 24, 48, 72, and 96 h post-infection (hpi). Genomic DNA was eliminated using the RQ1 RNase-free Dnase, and 600 ng of the resulting DNA-free RNA was retrotranscribed using random hexamers primers (Thermo Scientific, San Jose, CA, USA), according to the manufacturer’s instructions. qPCR was then performed using IFN-α, Mx, and ISG15 specific primers (Table 3) under the following cycling conditions: 3 min of hot start at 95 °C, followed by 40 cycles of 5 s at 95 °C, and 10 s at 60 °C. The ribosomal 18S RNA was used as the housekeeping gene [34], and the fold-change was calculated according to the method described by Pfaffl 2001 [35].

For poly I:C stimulation, we added 2 mL of L-15 media 10% FBS supplemented with 20 µg/mL poly I:C. Timepoints collection and processing were performed as described above.

### 2.12. Computer Analysis

Sanger sequencing results were analyzed by performing a BLAST search using the server’s default parameters to identify the sequence with the highest identity (>99%). This sequence was then used to perform a multiple sequence alignment against the corresponding pSS-URG plasmid (Table 2). Multiple sequence alignments were performed using ClustalW version 1.81 using the server’s default parameters.

### 2.13. Statistical Analysis

The data presented in this work is expressed as the average standard error of the mean (SEM) of at least three independent experiments. Statistical analysis involves the non-parametric Mann–Whitney U two-tailed test (α = 0.05) and the Pearson correlation test (α = 0.05). For both tests, a *p*-value below 0.05 was considered statistically significant. 

All data analyses were performed using GraphPad Prism software, version 6.0 (GraphPad Software, San Diego, CA, USA).

## 3. Results

### 3.1. Rescue of Recombinant Reassortant ISA Virus

**Figure 1:**
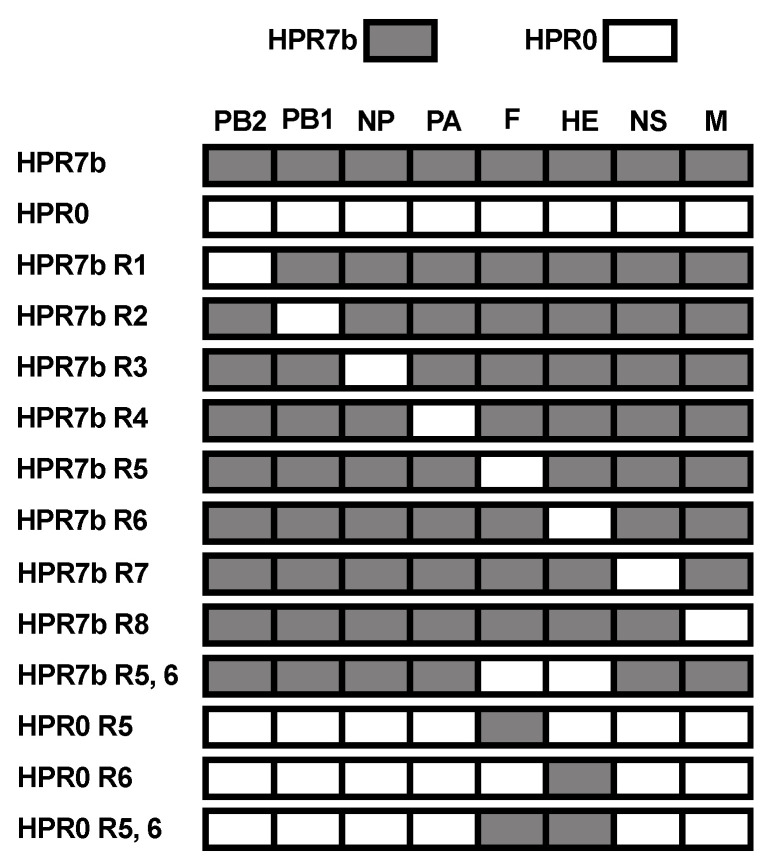
Genetic arrangement of reassortant viruses produced in this study. Genetic composition of each virus generated in this work. Grey denotes HPR7b segments, and white represents HPR0 segments. PB2: Segment 1; PB1: Segment 2; NP: Segment 3; PA: Segment 4; F: Segment 5; HE: Segment 6; NS: Segment 7; M: Segment 8. Each name refers to the genetic backbone, HPR7b or HPR0, and the “R” refers to the reassorted segment.

A total of 14 recombinant reassortant viruses were generated in this work. Of these 14 viruses, two contained the full genetic backbone from either an HPR7b (ISAV 752_09) or an HPR0 (SK779/06) isolate (Figure 1). These viruses served as controls to determine changes in replication, cytopathicity, and innate immune gene induction in the reassortant constructs.

A total of eight viruses were single reassortants, and they were generated using an HPR7b backbone and exchanging one segment for its HPR0 homolog. We also generated one virus with an HPR7b backbone containing both segments 5 and 6 of an HPR0 genotype. We also rescued two viruses with an HPR0 backbone carrying either segment 5 or 6 of an HPR7b genotype. Finally, a virus containing both segments 5 and 6 from HPR7b and the six remaining segments from HPR0 was produced.

**Figure 2:**
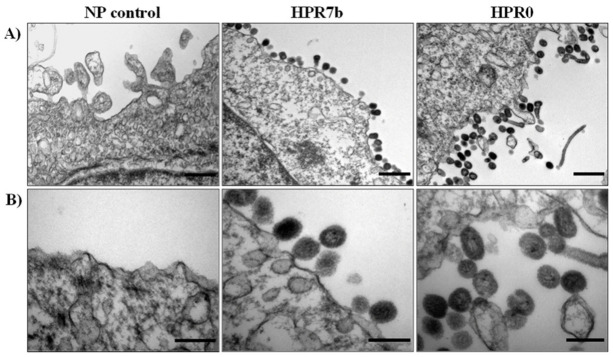
Viral particle morphology of rescued rISAV. EM imaging of ASK cells infected with either rISAV-HPR7b or rISAV-HPR0 at 4 dpi. (**A**) Representative transmission electron micrographs of ASK cells incubated with supernatants from NP control passage 3, rISAV-HPR7b, and rISAV-HPR0. Line in B panel: 500 nm. (**B**) Magnification of (**A**) images. Line in A panel: 200 nm.

After transfection, the supernatant was passed three times in ASK cells, and the inoculum from the first- and third-blind passage was tested for viral infection by endpoint RT-PCR targeting segment 6. All tested supernatants from infected cells showed a PCR product of the expected size in passages 1 (data not shown) and 3 (Figure S1), while no PCR product was obtained from the NP control. To confirm this result, ASK cells were infected with the control viruses (rISAV-HPR7b and rISAV-HPR0), and were observed under the electron microscope (Figure 2). As expected, no viral particles were seen in the cells incubated with the NP control supernatant. On the other hand, when cells were infected with either rISAV-HPR7b or rISAV-HPR0 inoculum, viral-like particles of ~100 nm in diameter morphologically consistent with ISAV were detected. 

### 3.2. rISAV Infection in ASK Cells

**Figure 3:**
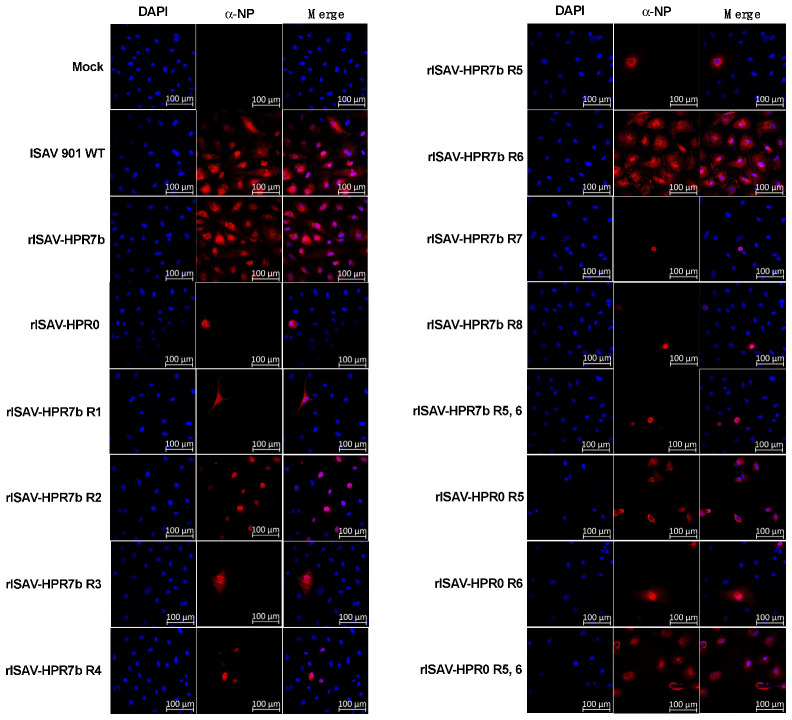
rISAV-NP protein detection by immunofluorescence in infected ASK cells. ASK cells were infected with each rISAV, incubated for five days, and analyzed by immunofluorescence for NP detection using a monoclonal anti-NP primary antibody and an anti-mouse Alexa Fluor 594-conjugated secondary antibody (red). Cellular DNA in the nucleus was stained with DAPI (blue). Non-infected cells and ISAV 901-infected cells are included as controls of NP expression. Scale Barr: 100 µM.

After confirming the rescue of reassortant viruses to be ultrastructurally consistent with isavirus, we analyzed if they were infectious in Atlantic salmon ASK cells. To this end, NP protein expression in infected cells was analyzed by indirect immunofluorescence at 5 dpi. This assay showed that cells infected with all viruses, including rISAV-HPR0, expressed NP (Figure 3), and, as expected, no NP-expressing cells were detected in uninfected control cells. These results confirm the infectious character of the supernatant containing the generated reassortant isavirus. 

After confirming the infective capacity of the reassortant viruses, we confirmed their genetic backbone and the sequence of the rearranged segments through Sanger sequencing. Sequences from all the viruses in passage 1 showed the expected backbone and rearranged genotype (Table S1), and the corresponding exchanged segments were in concordance with the plasmids used for the rescue.

### 3.3. Multiple Segments Determine ISAV Replicative Phenotype

**Figure 4:**
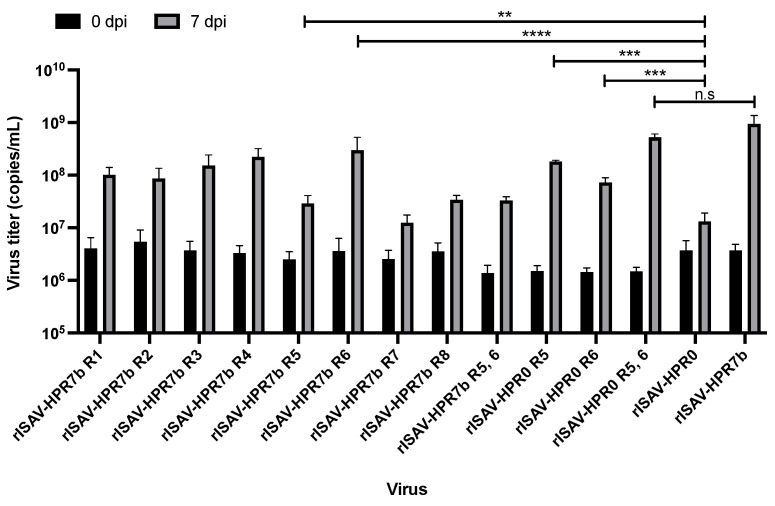
rISAV viral titers obtained in infected ASK cells at 0 and 7 dpi. ASK cells were infected at an MOI of 0.1, and the segment 8 copy number for each virus from the supernatant was determined by qRT-PCR at 0 and 7 dpi. Viral titers ± SEM from three independent experiments. α = 0.05; ** *p* < 0.005; *** *p* < 0.0005; **** *p* < 0.00005. n.s. not significant.

To analyze the replicative phenotype of each reassortant, ASK cells were infected at an MOI of 0.1, and viral titers from the supernatant of infected cells were determined by qRT-PCR at 0, 3, 5, and 7 dpi. Over time, the segment 8 copy number increase confirms that all reassortant viruses could infect and replicate in ASK cells. Moreover, this data revealed that the most replicative virus was rISAV-HPR7b, while the least was rISAV-HPR0 (Figure S2). Most viruses showed a substantial increase in the viral titer at 3 dpi, with significant differences between reassortants at 5 dpi. At 7 dpi, maximum virus titers for each virus were achieved. Surprisingly, the reassortment of all segments in an HPR7b (ISAV 752_09) backbone with its HPR0 (SK779/06) homologous segments negatively affected the replicative capacity of the viruses. The virus rISAV-HPR0 had the lowest titer, with only 1.3 × 10^7^ copies/mL (Figure 4), and a similar result was obtained with both rISAV-HPR7b R5 and rISAV-HPR7b R7. Interestingly, segment 6 appeared to have a minimal effect on this characteristic, since the virus rISAV-HPR7b R6 showed a slight decrease in the segment 8 copy number at 7 dpi, and the rISAV-HPR0 R6 virus exhibited a modest increase. Nonetheless, a dramatic decrement in the viral titer at 7 dpi was observed when an HPR0 segment 5 was introduced in an HPR7b backbone (rISAV-HPR7b R5). On the other hand, the virus rISAV-HPR0 R5 showed a titer of 1.8 × 10^8^ copies/mL being one log greater than the rISAV-HPR0 titer. Finally, a cooperative effect between segments 5 and 6 was observed, noted as an increase in the titer of the rISAV-HPR0 R5, 6 virus, which did not present statistical differences with rISAV-HPR7b. 

As a control, we performed infection kinetics in the presence of 0.5 µg/mL of ribavirin to ensure that viral titer increase over time was only due to virus replication, and not related to other factors (i.e., plasmids or defective particles). This experiment showed that most viruses could not replicate under this condition (Figure S3), since viral titers recorded at 0 dpi did not change at 7 dpi.

### 3.4. Multiple Segments Determine the Host Range of ISAV In Vitro

**Figure 5:**
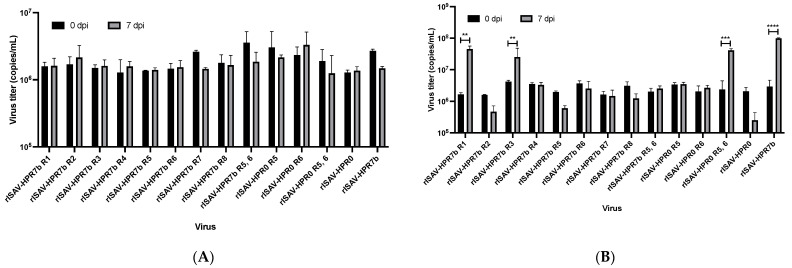

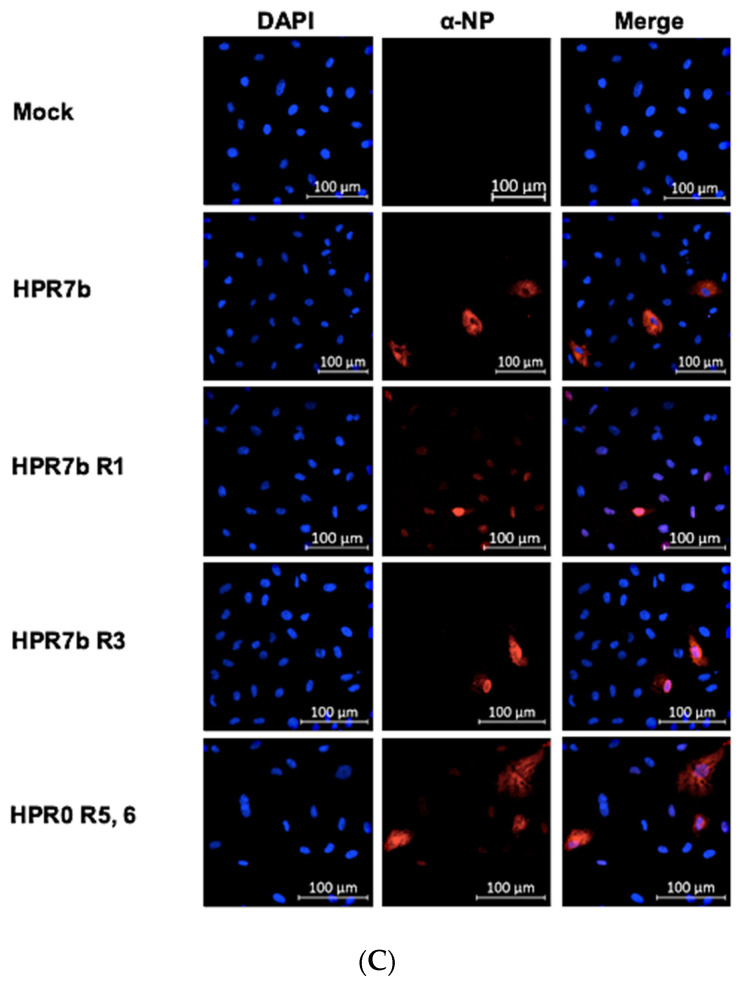
rISAV infection assays in RTG-2 and CHSE-214 cells. RTG-2 and CHSE-214 cells were infected at an MOI of 0.1, and viral titers were recorded at 0 and 7 dpi by qRT-PCR. Viruses showing replication activity in CHSE-214 cells were assessed via indirect immunofluorescence. (**A**) Infection kinetics of reassortant viruses in RTG-2 cells at 0 dpi (black) and 7 dpi (grey). (**B**) Infection kinetics of reassortant viruses in CHSE-214 cells at 0 dpi (black) and 7 dpi (grey). (**C**) Immunofluorescence detection of ISAV NP protein in infected CHSE-214 cells that were incubated with each virus showing replicative activity in (**B**); NP viral protein was detected at 5 dpi using a monoclonal anti-NP primary antibody and an anti-mouse Alexa Fluor 594-conjugated secondary antibody (red). Cellular DNA was stained in the nucleus with DAPI (blue). Uninfected control cells are included (mock). Viral titers ± SEM from three independent experiments. α = 0.05; ** *p* < 0.005; *** *p* < 0.0005; **** *p* < 0.00005. Scale Barr: 100 μM.

The replicative capacity of reassortant viruses in multiple hosts was assessed by infecting cell lines derived from different salmonid species, such as RTG-2 (Rainbow trout) and CHSE-214 cells (Chinook salmon). To this end, both cell lines were infected at an MOI of 0.1, and supernatants were collected at 0, 3, 5, and 7 dpi for further viral segment 8 quantitation via qRT-PCR. The viral titer in the supernatant of infected RTG-2 cells did not increase at any assessed time point, since the viral titer recorded at 7 dpi was the same or less than the titer at 0 dpi (Figure 5A), revealing that all reassortant isaviruses could not replicate in this rainbow trout-derived cell line.

In contrast, when salmon Chinook CHSE-214 cells were infected, viruses rISAV-HPR7b, rISAV-HPR7b R1, rISAV-HPR7b R3, and rISAV-HPR0 R5, 6 exhibited an increase in the viral titer by two orders of magnitude at 7 dpi when compared with the titer recorded at 0 dpi (Figure 5B). The rest of the viruses did not show replication in CHSE-214 cells. To confirm the result obtained by qRT-PCR, we performed indirect immunofluorescence that revealed NP expression in infected CHSE-214 cells (Figure 5C) when infected with the rISAV-HPR7b, rISAV-HPR7b R1, rISAV-HPR7b R3, and rISAV-HPR0 R5, 6 viruses. In contrast, no NP-expressing cells were observed when the remaining viruses were used to infect CHSE-214 cells (data not shown).

### 3.5. The Cytopathic Effect Induced by ISAV Is Highly Impacted by Segment 5 Genotype

**Figure 6:**
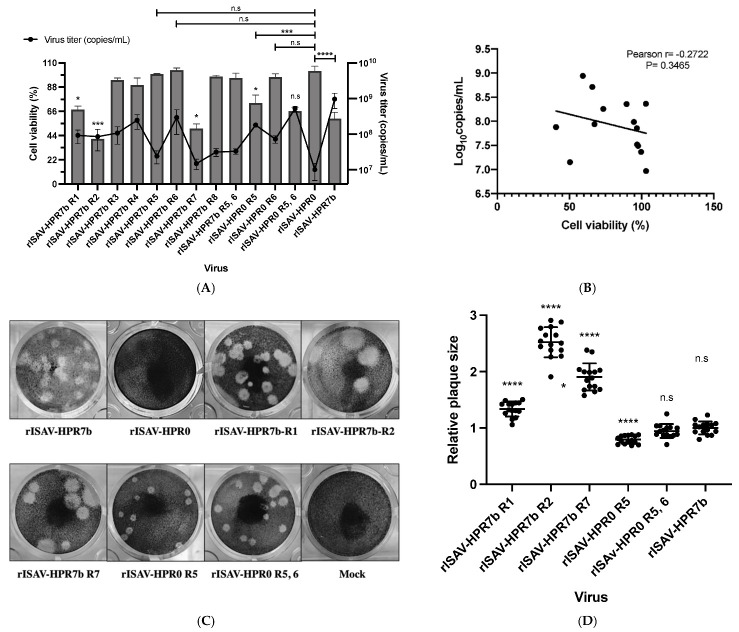
Effect of ISAV segments rearrangement on cell viability and cytopathic effect induction in ASK cells. (**A**) Quantification by MTT assay of cellular viability at 7 dpi (left Y-axis) of ASK cells infected at an MOI of 0.1, contrasting with viral titer (right Y-axis, black line) from infected cells supernatants determined by segment 8 absolute quantitation. (**B**) Pearson correlation analysis of cell viability and viral titer at 7 dpi using the data of (**A**). (**C**) Representative photographs of plaques produced by rISAV in ASK cells at 18 dpi. (**D**) Relative plaque size of the recombinant viruses compared to those produced by rISAV-HPR7b. Cellular viability and viral titer ± SEM from three independent experiments. α = 0.05; * *p* < 0.05; *** *p* < 0.0005; **** *p* < 0.00005; n.s. not significant.

The ability of reassortant ISA viruses to produce a cytopathic effect in ASK cells was assessed by infecting cells at an MOI of 0.1, and performing a cellular viability assay (MTT assay) at 7 dpi. In this assay, cells infected with the virus rISAV-HPR7b showed a cell viability of 59.2% (Figure 6A). Interestingly, cells infected with either rISAV-HPR7b R2 or rISAV-HPR7b R7 showed strongly reduced cell viability of 40.9% and 50.5%, respectively. On the other hand, cells infected with the virus rISAV-HPR0 presented a 100% viability at 7 dpi, as was also the case of cells infected with viruses rISAV-HPR7b R3, rISAV-HPR7b R4, rISAV-HPR7b R5, rISAV-HPR7b R6, rISAV-HPR7b R8, and rISAV-HPR7b R5, 6. Still, a substantial reduction in the cell viability was detected when segment 5 of an HPR7b genotype was introduced in an HPR0 backbone (rISAV-HPR0 R5). However, the introduction of segment 6 to an HPR7b genotype in an HPR0 backbone (rISAV-HPR0 R6) did not appear to induce cell death. On top of that, the virus rISAV-HPR0 R5, 6 did not show statistically significant differences with the cell viability of cells infected with rISAV-HPR7b, and the virus rISAV-HPR7b R5, 6 did not show differences with rISAV-HPR0 either.

In addition to cell viability, we also determined the segment 8 copy number from the supernatant of infected cells at 7 dpi to analyze the relationship between the replicative capacity of the virus and cell viability. A Pearson correlation test (Figure 6B) showed no correlation among these variables (r = −0.2722). 

As a control, we performed 0.5 µg/mL of ribavirin infections to ensure that cell death was only due to virus replication. This experiment demonstrated that infected cells showed 100% cell viability at 7 dpi when infections were incubated with ribavirin (Figure S4). 

The severity of the cytopathic effect was assessed by analyzing the size and morphology of plaques produced by each virus. To do this, ASK cells were infected with ten-fold serial dilutions of each inoculum, and incubated for 18 days. In this assay, only the viruses rISAV-HPR7b, rISAV-HPR7b R1, rISAV-HPR7b R2, rISAV-HPR7b R7, rISAV-HPR0 R5, and rISAV-HPR0 R5, 6 demonstrated the ability to produce plaques (Figure 6C), and no plaques were observed in cells infected with rISAV-HPR0. The rest of the viruses assayed in 6A did not produce plaques (data not shown). Regarding the plaque-forming viruses, rISAV-HPR7b R1 showed plaques 1.3 times larger than rISAV-HPR7b (Figure 6D), and interestingly, the viruses rISAV-HPR7b R2 and rISAV-HPR7b R7 produced the largest plaques, being 2.5 and 1.9 times larger, respectively, than those produced by rISAV-HPR7b. Furthermore, the introduction of segment 6 of an HPR7b genotype in an HPR0 backbone (rISAV-HPR0 R6) did not enable the virus to produce plaques, although the virus rISAV-HPR7b R6 lost this ability. Finally, the introduction of segment 5 of an HPR7b genotype, but not segment 6, in an HPR0 backbone made the virus produce smaller plaques than rISAV-HPR7b, and when both segments were exchanged, the virus rISAV-HPR0 R5,6 produced plaques of the same size as rISAV-HPR7b.

### 3.6. Segment 5 Is Involved in the Late Steps of Virus Adsorption and Uptake

Since segments 5 and 6 are involved in the early steps of the viral cycle, we investigated if their genotype has implications on these processes. To assess the effect of the reassortment, ASK cells were infected at an MOI of 1, and then incubated for 1 or 2 h at 15 °C. Afterward, cellular RNA was extracted, and the virus titer was determined by qRT-PCR. 

**Figure 7:**
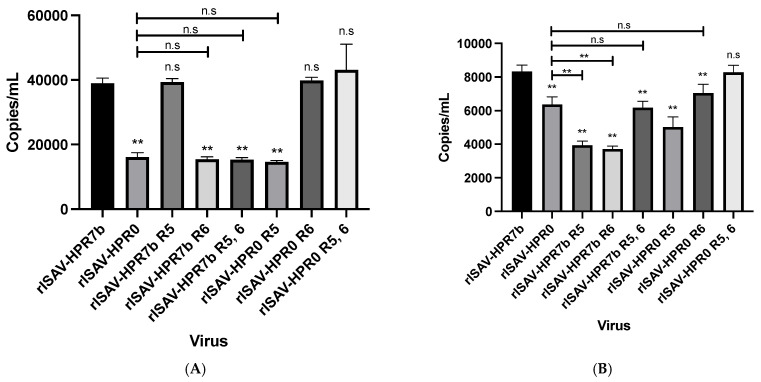
rISAV quantification assay to determine the effect of segments 5 and 6 genotype on the early and late steps of adsorptions and uptake. (**A**) Absolute quantification of ISAV segment 8 copy number of the isavirus attached, and uptake at 1 hpi from ASK cells infected at an MOI of 1. (**B**) Absolute quantification of isavirus segment 8 copy number at 2 hpi from washed ASK cells infected at an MOI of 1. Copies/mL ± SEM from three independent experiments. α = 0.05; ** *p* < 0.005; n.s. not significant.

After one hour of incubation, viruses carrying segment 6 of an HPR7b genotype showed a higher titer (~40,350 copies/mL) than those carrying an HPR0 segment 6 (Figure 7A). On top of that, viruses carrying either segment 5 of an HPR7b or an HPR0 genotype did not show differences compared with their backbone controls, demonstrating that segment 6, but not segment 5, is implicated in the early stages of adsorption and uptake. However, after two hours of incubation, all the viruses carrying segments 5 and 6 of the same genotype had the highest titers. Moreover, the virus rISAV-HPR7b showed a titer of 8329 copies/mL, which is not statically different from rISAV-HPR0 R5, 6 titers (Figure 7B). On the other hand, the viruses with segments 5 and 6 of different genotypes had an average titer of 4082 copies/mL, which represents a drop of 50% in the viral titer compared with their backbone controls. Taken together, these results suggest that only segment 6 is involved in the early stages of viral adsorption and uptake. Still, segments 5 and 6 are implicated in the late steps of these processes.

### 3.7. Recombinant Viruses Induce Distinct Cytokine Expression Profiles Depending on Their Genetic Composition

**Figure 8:**
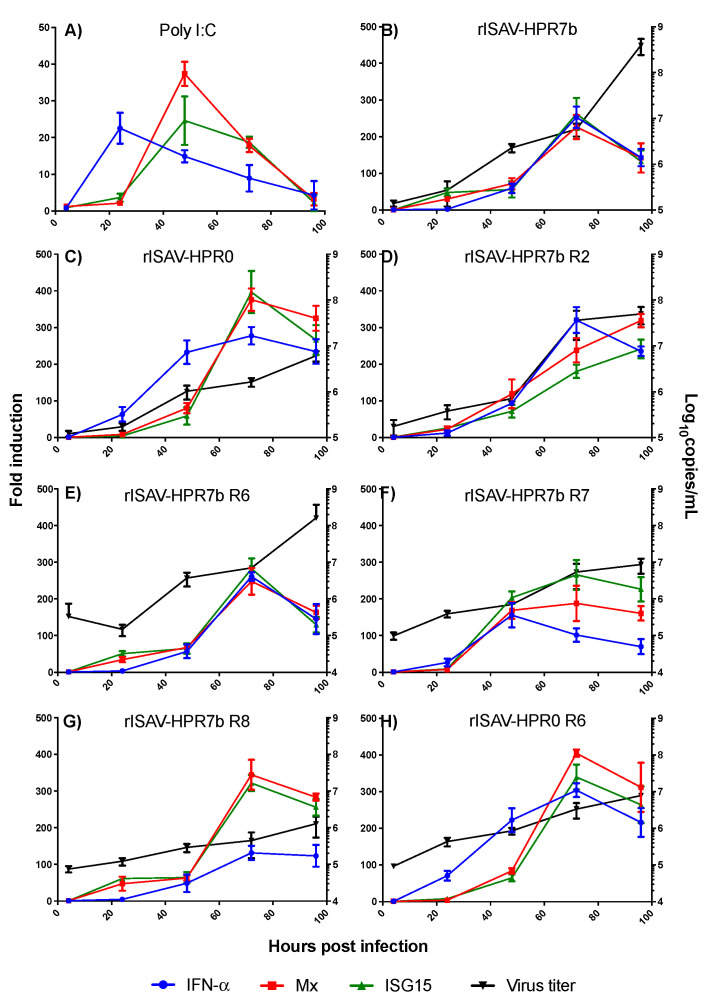
IFN-α, Mx, and ISG15 expression profile induced by reassortant rISAV infection in ASK cells. Relative gene expression (left Y-axis) of IFN-α (blue), Mx (red), and ISG15 (green) genes in ASK cells following rISAV infection. Gene expression and viral titer (right Y-axis, black) were measured at 4, 24, 48, 72, and 96 hpi. (**A**) Expression profile induced by 20 µg/mL poly I:C treatment. (**B**) Expression profile induced by rISAV-HPR7b infection. (**C**) Expression profile induced by rISAV-HPR0 infection. (**D**) Expression profile induced by rISAV-HPR7b R2. (**E**) Expression profile induced by HPR7b R6. (**F**) Expression profile induced by rISAV-HPR7b R7. (**G**) Expression profile induced by rISAV-HPR7b R8. (**H**) Expression profile induced by rISAV-HPR0 R6. Fold induction and viral titers ± SEM from two independent experiments.

To conclude the characterization of the reassortant viruses of this study, we analyzed the cytokine expression profile induced by some of them, especially those where segments related to immune response modulation previously described in either ISAV or Influenzavirus A were reassorted. To do so, ASK cells were infected with either rISAV-HPR7b, rISAV-HPR0, rISAV-HPR7b R2, rISAV-HPR7b R6, rISAV-HPR7b R7, rISAV-HPR7b R8, and rISAV-HPR0 R6 at an MOI of 0.1, and then cellular RNA was extracted at 4, 24, 48, 72, and 96 hpi to quantify the mRNA transcript level of the IFN-α, Mx and ISG15 genes by qRT-PCR (Figure 8, Table 4). 

As a control of the cellular immune response, we incubated ASK cells with 20 µg/mL poly I:C (Figure 8A). Under this condition, a rapid and transit upregulation of the analyzed genes was observed. After 24 h, the maximum expression level of IFN-α (21-fold) was achieved, followed by a steady decrease. On the other hand, the maximum expression of Mx and ISG15 was observed at 48 h with 37- and 23-fold changes, respectively. Cells infected with rISAV-HPR7b showed the maximum expression at 72 h (Figure 8B) with an average of 246-fold change for each gene. The virus rISAV-HPR0 showed a similar pattern, reaching a peak of expression at 72 hpi with nearly 400-fold changes for each gene (Figure 8C). Nonetheless, IFN-α showed a rapid expression, as early as 24 hpi. On the other hand, the virus rISAV-HPR7b R2 (Figure 8D) induced the maximum expression of IFN-α at 72 hpi, while the expression of other genes continued increasing until 96 hpi. Interestingly, this virus showed a marked reduction in the expression of ISG15 (180.5-fold), compared with both rISAV-HPR7b and rISAV-HPR0 (Table 2). Quite the opposite, the virus rISAV-HPR7b R6 showed the same expression pattern as rISAV-HPR7b (Figure 8E), with only slight differences in the fold change that was not statistically significant. When the virus rISAV-HPR7b R7 was analyzed, a dramatic reduction of IFN-α expression 

Was detected (Figure 8F), reaching the maximum expression at 48 hpi with only 155-fold changes. In the cases of Mx and ISG15, the maximum expression was at 72 hpi, with 188- and 265-fold changes, respectively. Similarly, the virus rISAV-HPR7b R8 reduced the expression of IFN-α to only 131-fold changes on average at 72 hpi (Figure 8G). Nonetheless, an immediate increase in Mx and ISG15 expression was observed as early as 24 hpi. Finally, the expression profile induced by the virus rISAV-HPR0 R6 was not significantly different from that observed with rISAV-HPR0 (Figure 8H). 

Parallel to this, the viral titer in the supernatant at each time point was measured by qRT-PCR. In all cases, the decay in the expression of the analyzed genes coincided with the highest number of viral titers registered, except for the virus rISAV-HPR7b R8, which did not show variation in the IFN-α expression between 72 and 96 hpi.

## 4. Discussion

Infectious salmon anemia virus has been a relevant problem for the fish farming industry due to some strains’ high virulence and infective capacity, such as the HPR7b genotype [38]. Moreover, in recent years the variant ISAV HPR0 has had a prevalence of 32% in Chilean farm-raised Atlantic salmon [39], increasing the risk of reassortment with a virulent strain that can result in the rise of a new reassortant virus with unknown phenotype. Due to role of most viral segments in the infective phenotype of isavirus remains unknown, this work used a reverse genetic approach to generate reassortant viruses by introducing segments of two prototype viruses, ISAV752_09 (HPR7b) and SK779/06 (HPR0), into each other’s backbone, and different infective properties of the resulting viruses were analyzed.

The successful recovery of synthetic ISA viruses was confirmed by transmission electron microscopy (TEM), in which the viruses observed showed morphologies consistent with the previously described wild-type virus regarding size and ultrastructure [29,40,41]. We also performed indirect immunofluorescence to detect the viral NP protein in infected cells, confirming that all reassortant viruses could infect ASK cells. The NP cellular localization was consistent with what was described by Aspehaugh et al., 2004 [42]. Taken together, these results showed that reassortant viruses were successfully obtained, achieving for the first time a viral culture of the complete HPR0 genotype. However, it should be noted that its 5′ and 3′ UTR ends were derived from an ISAV 752_06 isolate due to the lack of HPR0 sequences. The HPR0 rescue highlights the importance of the 5 and 3′ UTR ends of the viral genome segments, which have been given little to no significance as virulence markers of isavirus. Moreover, in the influenza A virus, UTR regions are associated with the replicative capacity, transcription of the viral genome, and also with the cap snatching process [43,44,45], and, given that isavirus isolates present a variable portion in their 3′ and 5′ [46], it could produce replication and transcription differences between isolates.

Before the characterization of the infective phenotype, the synthetic reassortant viruses were genetically characterized to confirm the rearrangement and segments 5 and 6 genotype, since it has been shown that ISAV can modify its HPR region in vivo [16]. We have also described this phenomenon with our reverse genetics system in vitro [30]. However, we have demonstrated that it is possible to obtain genetically stable viruses via at least three passages with this system. Using the supernatant of the first blind passage and Sanger sequencing, we determined that all the viruses had the expected genotypes. These well-characterized viruses were subsequently used to evaluate the infective phenotype, ensuring that all the infectivity differences are solely the result of segment reassortment. 

The first infective characteristic evaluated was the replicative capacity of the reassortant viruses in ASK cells, revealing that all HPR0-derived segments decrease the maximum viral titer obtained at 7dpi in an HPR7b backbone. Moreover, this effect was exacerbated with the replacement of segments 5, 7, and 8, and a dual reassortment of segments 5 and 6. This phenomenon has been previously described in influenza A viruses, in which the proteins coded by the HA and NA segments seem to work together, since the NA protein modulates the ability of HA to bind the cellular receptor [47], and modify the post-translational processing of HA [48]. The results obtained in this work suggest that a similar effect occurs with isavirus, in which early studies had shown that the HE and the F protein work together during the membrane fusion events after virus uptake [21], since HE controls the efficiency of the fusion protein cleavage. Therefore, it cannot be ruled out that the fusion protein influences HE activity, as described in influenza A. 

On the other hand, the effects of the reassortment of segments 7 and 8 could be due to the interaction between M1 and NEP to promote the nuclear export of the vRNPs to the cytoplasm, something that has been described in influenza A viruses [49,50]. This antecedent can explain that mutations in both segments affect the interaction of the encoded proteins with host elements, and therefore reduce the efficiency of the vRNPs export [51]. Although only a few studies about this interaction have been published for ISAV, more experiments are required to determine the underlying mechanisms of segments 7 and 8 reassortment. 

Analysis of the capacity of the reassortant viruses to replicate in distinct salmonid cell lines showed that segments 1, 3, and together 5 and 6, are involved in the ability of the virus to infect and replicate in CHSE-214 cells. To date, it has only been determined that not all ISAV strains can replicate in CHSE-214 cells, probably due to variations in the highly polymorphic region of the HE protein [32]. Still, no other segments have been associated with this property. However, in influenza A, the host range is mainly determined by the HA and NA features, as well as the affinity of some viral proteins for importin-α [26,52]. In this line, point mutations in PB2, PB1, PA, and NP affect, among other things, their interaction with this cellular protein [53], impairing their translocation into the nucleus, and therefore preventing the viral genome replication and transcription. We propose that something similar occurs in ISAV, since all segments implicated in CHSE-214 infection code for at least one protein with nuclear localization (except HE and F). This hypothesis can also explain any virus replicated in RTG-2 cells, since identities between importin-α peptide sequence from *Salmo salar* and *Oncorhynchus mykiss* do not exceed 57%, while identities between *Salmo salar* and *Oncorhynchus tshawytscha* are close to 90% (data not shown). 

On the other hand, the underlying mechanism involved in the replication of segments 5 and 6 reassortants in CHSE-214 cells might be again an interaction of the two proteins encoded. In the case of the virus rISAV-HPR0 R6, the deleted HPR region exposes the fusion protein cleavage sites. Still, as segment 5 is an HPR0, it does not have the IN4 insertion, which theoretically provides additional cleavage sites, meaning that activation of the protein is not possible. Quite the opposite, the virus rISAV-HPR0 R5 has the IN4 insertion in segment 5 [13]. However, the full-length HPR region hides them. Thus, only a virus carrying both HE and F genes of compatible genotypes will be able to infect CHSE-214 cells successfully. 

The analysis of the cytopathic effect induced by rISAV infection showed that most segments were related to this characteristic, although segments 2 and 7 dramatically reduce cellular viability. This result was surprising, considering that segment 7 is one of the most conserved among the ISAV genome, with only a few nucleotide mutations, and most of them are silent mutations [2]. In 2004, it was shown that ISAV induces necrosis and apoptosis differentially depending on the infected tissue, necrosis being triggered by proteins encoded by segment 7 and/or segment 1 [54]. This previous work, along with our results, suggests that the reduced cellular viability produced by rISAV-HPR7b R7 infection could be the result of strengthening the pro-apoptotic or pro-necrotic activity of the genetic composition of the virus. Nevertheless, the precise mechanism of this phenomenon should be determined experimentally in further studies.

On the other hand, the plaque assay outcome was consistent with cellular viability results, with rISAV-HPR7b R2 and rISAV-HPR7b R7 viruses producing the largest plaques. Surprisingly, the virus rISAV-HPR0 R5 was able to produce plaques, indicating the induction of cell death solely by the exposure of cells to a fusion protein of a virulent origin, suggesting that the fusion protein triggers cell death per se, which could be an explanation to previous observations that some viruses with the same HPR sequence have different virulence degrees [2]. Something similar has been described for influenza A virus, where apoptosis can be induced by only the exposure of cells to the HA protein, which in influenza A also contains the fusion domain [55], supporting the idea that the F protein alone can trigger cell death. Additionally, we did not observe any correlation between the degree of cytopathic effect and the viral titer of the reassortant viruses, contrary to what was expected, meaning that cell death is more related to the genetic backbone of isavirus and their interaction with the host cells, rather than to its replication rate, representing an interesting topic for further studies. Assays with salmon might shed light on the actual effects of these reassortments on the pathology caused by the isavirus in vivo.

We found significant differences between the HPR7b and the HPR0 genotypes regarding the adsorption and uptake events. Our results suggest that only the HE protein participates in the early events of this process, contrasting with previous studies that did not find differences between the adsorption capacity of the HE protein of both HPR7b and HPR0 genotypes [14]. However, the referred study utilized a heterologous expression system in CHSE-214 cells to express the HE protein of both genotypes, since the HPR0 virus could not be cultivated; due to this, the work by McBeath et al., 2011 [14] did not consider interactions between HE and other viral proteins such as F, which our results strongly suggest modulates together different processes of the viral cycle. 

The last characteristic we evaluated to characterize the reassortant viruses was the cellular immune response induced by them, since previous evidence has shown that highly virulent ISA viruses induce poor expression of ISGs at the transcript and the protein levels. In contrast, viruses presenting low virulence induce an exacerbated expression [56]. The exchange of segment 8 of an HPR7b genotype for its HPR0 homolog increased the expression of the analyzed ISGs genes by 300-fold, near to expression levels induced by rISAVHPR0. Nonetheless, segment 2 exchange produced a decrease in the expression of the ISGs genes, similar to the effect observed with rISAV-HPR7b R7. This result correlates with plaque size. Large plaques can be due to a low or deficient expression of the antiviral genes allowing plaque growth [57]. However, this hypothesis fails to explain the rISAV-HPR7b R6 and rISAV-HPR7b R8 viruses’ behavior, since they did not produce plaques, but still induce similar expression kinetics as rISAV-HPR7b, which does produce plaques. This suggests that either the magnitude of the expression of the antiviral genes is not a determinant to preventing ISAV infection, but the timing of IFN-α expression, or that some viral proteins induce cell death directly, such as we previously mentioned is the case in Influenza A [55]. Nonetheless, since this hypothesis fails to explain the behavior of some viruses, more studies will be needed to fully understand this phenomenon. 

To conclude, with the results presented in this work, we demonstrate that most genomic segments contribute to different characteristics of ISAV infectivity. Our results show that segment 6 gene product participates in the early stages of adsorption and uptake, while segments 5 and 6 products participate together in the late stages of virus uptake. All segments seem to be involved in genome replication and/or transcription, segment 5 being one of the most important. We also showed that segments 2, 7, and 8 confer isavirus the capacity to modulate the cellular immune response, which might trigger cell death. Following this, we showed that the introduction of only segment 5 of an HPR7b genotype in an HPR0 backbone makes the virus able to produce cell death, meaning that this segment 5 has important implications in the infectivity of isaviruses, as was suggested recently by Ditlecadet et al., 2022 [58]. All of our results make it evident that reassortment among ISAV strains can represent a severe risk to the salmon industry, since segment 5 alone is sufficient for an HPR0 virus to acquire HPR7b-like virulent characteristics, making it necessary to include segment 5 on ISAV genomic surveillance.

## Figures and Tables

**Table 1:** GenBank accession number for the viral strains used for reverse genetics plasmids construction.

Plasmid	Cloned Segment	Segment Genotype	GenBank Accesion Number
pSS-URG-S1/1	1	HPR0	EU118815
pSS-URG-S2/1	2	HPR0	EU118816
pSS-URG-S3/1	3	HPR0	EU118817
pSS-URG-S4/1	4	HPR0	EU118818
pSS-URG-S5/1	5	HPR0	EU118819
pSS-URG-S6/1	6	HPR0	EU118820
pSS-URG-S7/1	7	HPR0	EU118821
pSS-URG-S8/1	8	HPR0	EU118822
pSS-URG-S1/2	1	HPR7b	GU830895
pSS-URG-S2/2	2	HPR7b	GU830896
pSS-URG-S3/2	3	HPR7b	GU830897
pSS-URG-S4/2	4	HPR7b	GU830898
pSS-URG-S5/2	5	HPR7b	GU830899
pSS-URG-S6/2	6	HPR7b	GU830900
pSS-URG-S7/2	7	HPR7b	GU830901
pSS-URG-S8/2	8	HPR7b	GU830902

**Table 2:** Primers used for ISAV detection and Sanger sequencing.

Segment	Name	Sequence	Reference
1	S1_Fw	CGGAACCAACTACCGAGGAG	This work
	S1_Rv	GGGATCTCCTCCTGTTTCAACT	
2	S2_Fw	TCAGGTGTATGCAGGGGAAAC	This work
	S2_Rv	TGAAGGCCCTCCAAGGTACT	
3	S3_Fw	GCTGCAGCAATCGAAAGGTC	This work
	S3_Rv	CGTCTTCATCAGCCAAAGCG	
4	S4_Fw	ACCAACGGGAAAGACAGAG	This work
	S4_Rv	TCAAGCCTCTCAGTTCCCA	
5	S5_Fw	CTGCGGAGGTACAACAGGTTA	This work
	S5_Rv	CTGGTACAGAATGGAACGGCA	
6	S6_Fw	GCCCAGACATTGACTGGAGATG	Cárdenas et al., 2020 [29]
	S6_Rv	GATGGTGGAATTCTACCTCTAGACTTGTA	
7	S7_Fw	TGTTTGTCACTGGCCCTGAG	This work
	S7_Rv	TGAGCCCGACAGGAAAGAAG	
8	S8_Fw	TGCTACTTACACTTGGCG	This work
	S8_Rv	TCTGCATCCTGCTGTGTAGC	

**Table 3:** Primers used for real-time qPCR analyses.

Gene	Name	Sequence	Reference
INF-α	Fw_IFN-α	GGACAAGAAAAACCTGGACG	Reyes-Cerpa et al., 2014 [36]
	Rv_IFN-α	CTTTCCTGATGAGCTCCCAC	
Mx	Fw_Mx	TGCAACCACAGAGGCTTTGAA	Haugland, Ø et al., 2005 [37]
	Rv_Mx	GGCTTGGTCAGGATGCCTAAT	
ISG15	Fw_ISG15	TGAAAAACGAAAAGGGCCAGAC	This work
	Rv_ISG15	CAGCCTCCCCTTAGACGGA	
18S	18S_Fw	GCAAATGCTTTCGCTTTCG	Jorgensen, S et al., 2006 [34]
	18S_Rv	TGTGCCGCTAGAGGTGAAATT	

**Table 4:** Fold induction of IFN-α, Mx, and ISG15 genes at 72 hpi. ASK cells were infected, and an MOI of 0.1 and cellular RNA were extracted at 72 hpi. Gene expression was determined by qRT-PCR using the 18S ribosomal gene as the housekeeping gene.

Fold Induction			
Virus	INF-α	ISG15	Mx
HPR7b	252.9	262.4	226.5
HPR0	277.4	396.9	376
HPR7b R2	320.4	180.5	238.3
HPR7b R6	261.1	282.6	247.9
HPR7b R7	155	265.7	188
HPR7b R8	131.5	322.1	344.5
HPR0 R6	304.2	339.6	404.8

## Data Availability

Not Applicable.

## Supplementary Materials

The following supporting information can be downloaded at: https://www.mdpi.com/article/10.3390/v14030631/s1, Figure S1: Detection of ISAV RNA from the supernatant of infected cells by RT-PCR; Figure S2: Kinetics of rISAV replication in infected ASK cells; Figure S3. Viral titers obtained at 0 and 7 dpi in ASK cells incubated with 0.5 µg/mL of ribavirin as antiviral; Figure S4: Effect of ribavirin treatment on rISAV-induced cell death; Table S1: Genotyping results of reassortant viruses by sequencing and bioinformatics analysis.
