# Supplementary material for: Infectious Salmon Anemia Virus Infectivity Is Determined by Multiple Segments with an Important Contribution from Segment 5
Source: Viruses. 2022 Mar 18;14(3):631. doi: 10.3390/v14030631 (PMC8954079; doi:10.3390/v14030631)
Supplement: Supplementary file 1 [file viruses-14-00631-s001.zip › viruses-1598900-supplementary.pdf]

viruses

MDPI

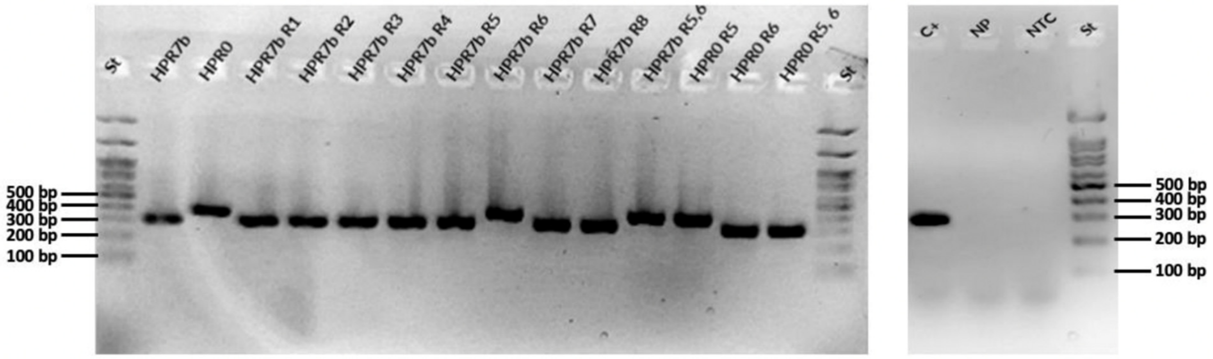

Figure S1. Detection of ISAV RNA from the supernatant of infected cells by RT-PCR. Detection of rISAV segment 6 was performed by RT-PCR from the supernatant of passage 3 at 7 dpi of the different reassortat viruses. RNA from supernatants of cell with Control NP and NTC was used in the RT-PCR. Two bands with different lengths were expected, which were HPR regions of HPR7b (312 bp)and HPR0 regions (382 bp). St, standard molecular size.

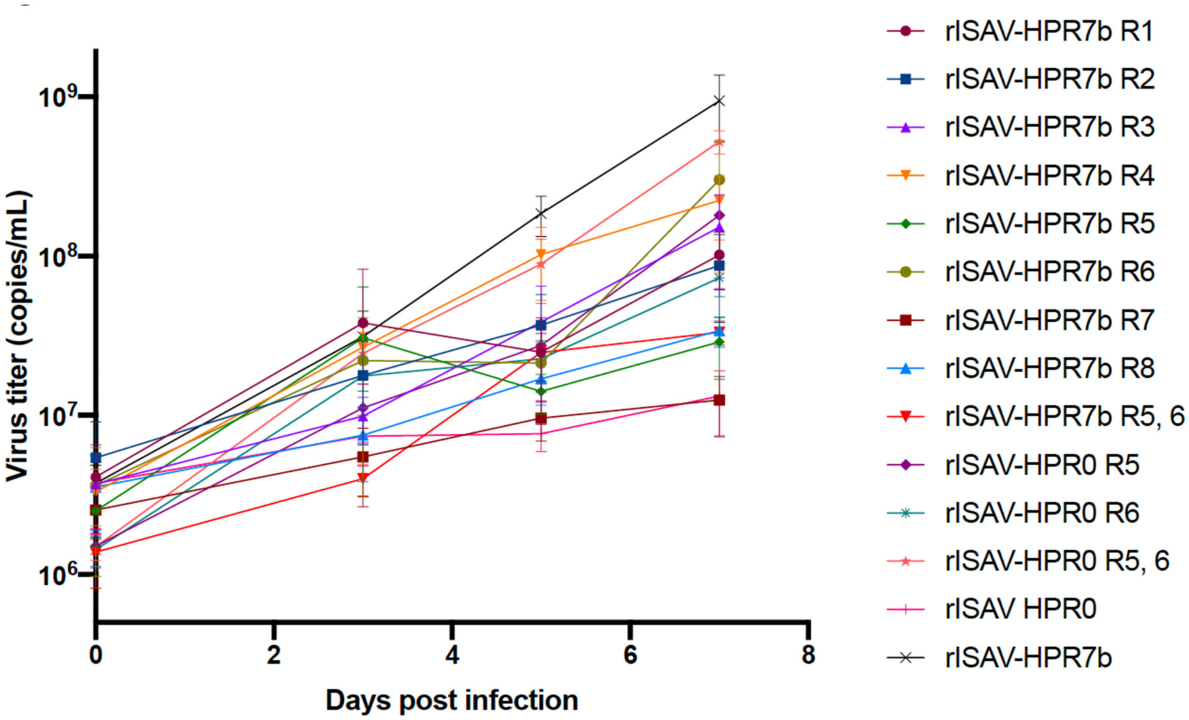

Figure S2. Kinetics of rISAV replication in infected ASK cells. ASK cells were infected at an MOI of 0.1 and the segment 8 copy number for each virus from the supernatant was quantified by qRT-PCR at 0, 3, 5, and 7 dpi. Viral titers ± SEM from three independent experiments.

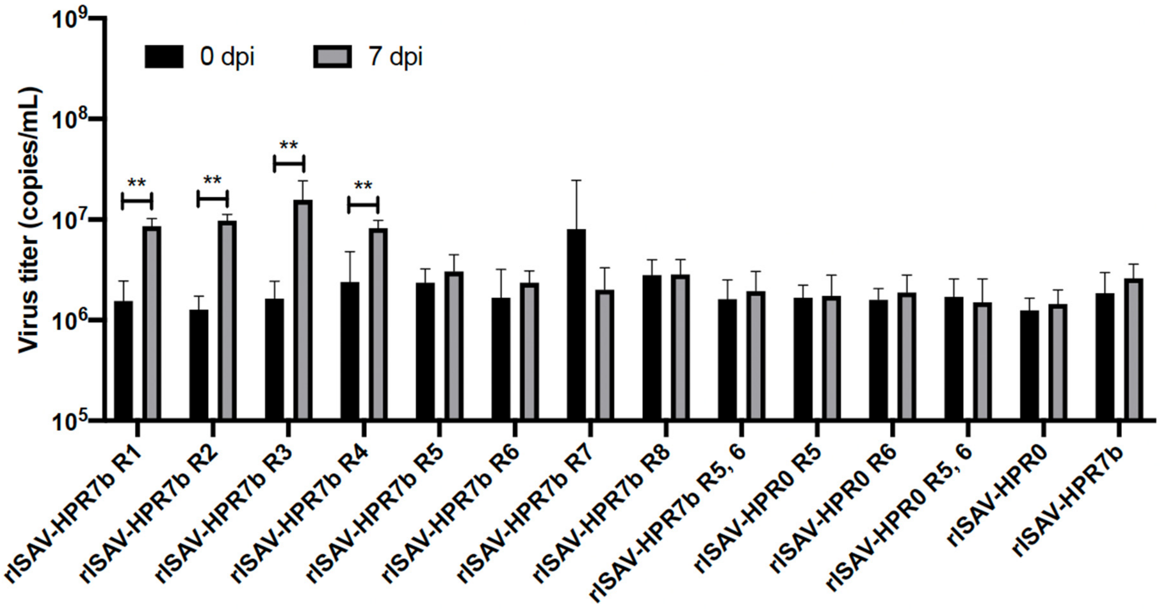

Figure S3. Viral titers obtained at 0 and 7 dpi in ASK cells incubated with 0.5 µg/mL of ribavirin as antiviral. ASK cells were infected at an MOI of 0.1 and after the 4-hour incubation period were supplemented with 0.5 µg/mL of ribavirin. The segment 8 copy number for each virus from the supernatant was determined by qRT-PCR at 0 and 7 dpi. Viral titers ± SEM from three independent experiments. α=0.05; **p ‹ 0.005.

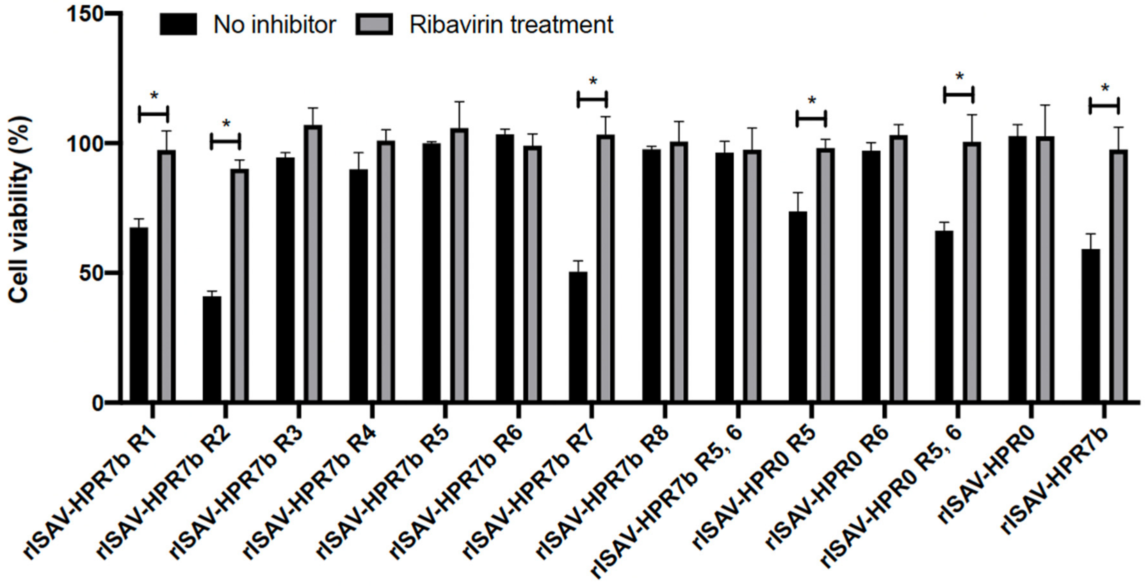

Figure S4. Effect of ribavirin treatment on rISAV-induced cell death. Quantification of cellular viability at 7 dpi (left Y-axis) of ASK cells infected at an MOI of 0.1 by MTT assay in the absence (black bars) or presence of 0.5 µg/mL of ribavirin (gray bars). Cellular viability ± SEM from three independent experiments. α=0,05; *p ‹ 0.05.

Table S1. Genotyping results of reassortant viruses by sequencing and bioinformatics analysis. The genotype of the rearranged segments (A) of each virus and the insertion region of segment 5 and the HPR region of segment 6 segments (B) are presented for each virus generated in this study.

	**A**	
**Virus**	**Reassorted segment**	**Genotype of reassorted segment**
**HPR7b R1**	1	HPR0
**HPR7b R2**	2	HPR0

**HPR7b R3**	3	HPR0
**HPR7b R4**	4	HPR0
**HPR7b R5**	5	HPR0
**HPR7b R6**	6	HPR0
**HPR7b R7**	7	HPR0
**HPR7b R8**	8	HPR0
**HPR7b R5, 6**	5 y 6	HPR0
**HPR0 R5**	5	HPR7b
**HPR0 R6**	6	HPR7b
**HPR0 R5, 6**	5 y 6	HPR7b
	**B**	
**Virus**	**Segment 5 genotype**	**Segment 6 genotype**
**HPR7b**	HPR7b	HPR7b
**HPR0**	HPR0	HPR0
**HPR7b R1**	HPR7b	HPR7b
**HPR7b R2**	HPR7b	HPR7b
**HPR7b R3**	HPR7b	HPR7b
**HPR7b R4**	HPR7b	HPR7b
**HPR7b R5**	HPR0	HPR7b
**HPR7b R6**	HPR7b	HPR0
**HPR7b R7**	HPR7b	HPR7b
**HPR7b R8**	HPR7b	HPR7b
**HPR7b R5, 6**	HPR0	HPR0
**HPR0 R5**	HPR7b	HPR0
**HPR0 R6**	HPR0	HPR7b
**HPR0 R5, 6**	HPR7b	HPR7b
